# A randomized hypofractionation dose escalation trial for high risk prostate cancer patients: interim analysis of acute toxicity and quality of life in 124 patients

**DOI:** 10.1186/1748-717X-8-206

**Published:** 2013-09-04

**Authors:** Darius Norkus, Agata Karklelyte, Benedikt Engels, Harijati Versmessen, Romas Griskevicius, Mark De Ridder, Guy Storme, Eduardas Aleknavicius, Ernestas Janulionis, Konstantinas Povilas Valuckas

**Affiliations:** 1 Department of Radiotherapy, Institute of Oncology, Vilnius University, Vilnius, Lithuania; 2 Department of Radiotherapy, UZ Brussel, Vrije Universiteit Brussel, Brussels, Belgium

## Abstract

**Background:**

The *α/β* ratio for prostate cancer is postulated being in the range of 0.8 to 2.2 Gy, giving rise to the hypothesis that there may be a therapeutic advantage to hypofractionation. To do so, we carried out a randomized trial comparing hypofractionated and conventionally fractionated image-guided intensity modulated radiotherapy (IG-IMRT) in high-risk prostate cancer. Here, we report on acute toxicity and quality of life (QOL) for the first 124 randomized patients.

**Methods:**

The trial compares 76 Gy in 38 fractions (5 fractions/week) (Arm 1) to 63 Gy in 20 fractions (4 fractions/week) (Arm 2) (IG-IMRT). Prophylactic pelvic lymph node irradiation with 46 Gy in 23 fractions sequentially (Arm 1) and 44 Gy in 20 fractions simultaneously (Arm 2) was applied. All patients had long term androgen deprivation therapy (ADT) started before RT. Both physician-rated acute toxicity and patient-reported QOL using EPIC questionnaire are described.

**Results:**

There were no differences in overall maximum acute gastrointestinal (GI) or genitourinary (GU) toxicity. Compared to conventional fractionation (Arm 1), GI and GU toxicity both developed significantly earlier but also disappeared earlier in the Arm 2, reaching significant differences from Arm 1 at week 8 and 9. In multivariate analyses, only parameter shown to be related to increased acute Grade ≥1 GU toxicity was the study Arm 2 (*p* = 0.049). There were no statistically significant differences of mean EPIC scores in any domain and sub-scales. The clinically relevant decrease (CRD) in EPIC urinary domain was significantly higher in Arm 2 at month 1 with a faster recovery at month 3 as compared to Arm 1.

**Conclusions:**

Hypofractionation at 3.15 Gy per fraction to 63 Gy within 5 weeks was well tolerated. The GI and GU physician-rated acute toxicity both developed earlier but recovered faster using hypofractionation. There was a correlation between acute toxicity and bowel and urinary QOL outcomes. Longer follow-up is needed to determine the significance of these associations with late toxicity.

## Background

External-beam radiotherapy (EBRT) has a long history of clinical use in the treatment of prostate cancer. There is a clear demonstration from conventionally fractionated radiotherapy dose escalation clinical trials of improved biochemical relapse-free survival rates with higher irradiation doses [1,2]. Conventionally fractionated dose escalation, however, results in treatment protraction, which could negatively impact patient’s lifestyle and possibly lowers biological benefit. Hypofractionation in the treatment of prostate cancer offers a shorter treatment course and increased convenience for patients [3-10].

There is increasing evidence that the α/β ratio for prostate cancer may be low and is in the range of 0.8 to 2.2 Gy [11]. If the prostate cancer *α/β* value is consistently lower than the appropriate values considered for late normal-tissue morbidity, significant increases in tumor control can be expected by changing from conventional fractionation to fewer larger fractions, without increasing the risk of acute and late toxicity. The fear of severe acute and late genitourinary (GU) and gastrointestinal (GI) toxicity has been an argument against hypofractionated dose escalation to the prostate and pelvic lymph nodes. However, planning studies have proved that IMRT enables the combination of better target coverage and sparing of the OARs [12]. The results to date support the conclusion that hypofractionated radiation therapy is relatively safe for the treatment of localized prostate cancer. No significant increase has been seen in the acute toxicity or the late adverse events as compared to standart dose regiments [3-6].

Most reports from prostate cancer hypofractionated EBRT studies have focused on physician-rated toxicities. Only some have used validated patient-reported quality of life (QOL) questionnaires [5-10]. We found QOL outcomes important because they are not well correlated with physician-related toxicities [13].

In this interim report we describe both, physician-related acute toxicities and patient-reported QOL measurements by validated Expanded Prostate Cancer Index Composite (EPIC), from the prospective randomized trial comparing hypofractionated and conventionally fractionated image guided IMRT (IG-IMRT) combined with androgen deprivation therapy for the first 124 high risk prostate cancer patients that were randomized in this study.

## Methods

### Patient population

Between January 2010 and May 2012, 124 consecutive patients were enrolled in this study. The inclusion criteria were as follows: histologically proven prostate adenocarcinoma; prostate-specific antigen (PSA) level ≤100 ng/ml; ECOG performance status <2; no evidence of distant metastases; no other malignancy except basal cell skin cancer; no contraindications for androgen deprivation therapy (ADT); no previous prostate surgery including transurethral resection; and most importantly, high risk features according to NCCN criteria: stage T3a-T3b, biopsy Gleason score (GS) of 8–10; pretreatment PSA level (iPSA) >20 ng/mL, or the presence of at least two of the following clinical characteristics: iPSA of 11–20 ng/ mL, T ≥2c, GS =7. Exclusion criteria included lymph node involvement and previous RT to the pelvis.

### Study design

The present Phase III, randomized trial design was formulated to test the hypothesis that compared with a conventional fractionation of 76 Gy in 38 fractions (5 fractions/week) at 2.0 Gy/fraction (Arm 1), the delivery of 63 Gy in 20 fractions (4 fractions/week) at 3.15 Gy/fraction (Arm 2, hypofractionation) to the prostate and seminal vesicles would be more effective while maintaining same rates of acute and late complications as well as patient-reported QOL changes. The pelvic lymph nodes were irradiated sequentially delivering 46 Gy at 2 Gy/fraction (Arm 1) or simultaneously – 44 Gy at 2.2 Gy/fraction (Arm 2). The hypofractionation regimen to the prostate and seminal vesicles was hypothesized to be equivalent to 84 Gy EQD_2_ assuming an *α/β* ratio of 1.5 Gy. The hypothesized difference of 8 Gy between Arm 1 and Arm 2 potentially results in a 15% gain in freedom from biochemical failure (FFBF) [14]. To detect this gain with 80% power and statistical significance (*p* < 0.05, two-sided), 120 patients were required in each group (240 total). With the regard to the pelvic lymph nodes both regimens hypothesized to be similar: 46 Gy (Arm 1) and 46.5 Gy (Arm 2) assuming an *α/β* ratio of 1.5 Gy. The protraction of hypofractionation regimen from 4 to 5 weeks (4 fractions/week) allowed to keep the acute mucosal time-corrected BED below 63 Gy_10_ limit to achieve tolerable acute reactions [15].

After institutional ethics board and Lithuanian Bioethics Committee approval the study started in 2010. The patients were enrolled and assigned to the study group with a balanced randomization method using a computer program. No blinding was possible. The study was scheduled to be completed within 5 years.

### Treatment

All patients were instructed to use Fleet enema in the morning of CT simulation day to empty the rectum, to urinate and drink 400 ml of water 30 minutes before CT simulation. CT simulation was performed in the supine position with knee and ankle fixing device. Patients were scanned with 2.5 mm thick slices from fourth lumbar vertebra to 3 cm below ischial bones. The entire prostate was outlined as CTV_p_, entire seminal vesicles as CTV_sv_. Pelvic lymph nodes were outlined as CTV_ln_ following RTOG consensus guidelines [16]. To obtain the planning target volume of the prostate (PTV_p_), the CTV_p_ was expanded in X, Y, and Z direction with a 10 mm margin, except posteriorly where a 7 mm margin was added. CTV_sv_ was expanded to PTV_sv_ with 10 mm in all directions, and CTV_ln_ to PTV_ln_ with a 5 mm margin. PTV_p_ and PTV_sv_ were merged into PTV1, PTV_ln_ - into PTV2. Rectum was outlined as a solid organ from rectosigmoid flexure down to the bottom of ischial bones. Bladder was also outlined as a solid organ. Large and small bowel were outlined as one structure, up to 10 mm above PTV2, encompassing the entire abdominal cavity.

All study patients underwent planning with Eclipse v. 10.0 (Varian Medical Systems, Palo Alto, CA) and sliding windows delivery. Treatment plans were generated using 5 to 7 co-planar photon beams of 6 MV. PTV1 and PTV2 were set to receive between 95% and 108%, clinical target volumes – between 99% to 108% of the prescription dose. Two sequential plans were made for Arm 1. Phase 1 contained 23 fractions of 2.0 Gy to PTV1 and PTV2, phase 2 contained an additional 15 fractions of 2.0 Gy to PTV1. Arm 2 patients were planned to treat with a simultaneously integrated boost (SIB), giving 20 fractions of 3.15 Gy to PTV1 and 20 fractions of 2.2 Gy to PTV2, using 4 fractions per week. With regard to OARs, both phases were combined in the Arm 1 plans and dose/volume restrictions were as following: rectum – maximum dose (Dmax) <80 Gy, volume receiving 55 Gy (V55) <50%, V70 <30%; bladder – Dmax <80 Gy, V55 <50%, bowel – Dmax <55 Gy, femoral heads – Dmax <50 Gy. Arm 2 plans had equivalent restrictions: rectum – Dmax <64.2 Gy, V50 <50%, V58 <30%; bladder – Dmax <64.2 Gy, V50 <50%, bowel – Dmax <50 Gy, femoral heads – Dmax <48 Gy.

Daily image guidance with kV cone beam CT (CBCT) with linac mounted on board imager (OBI - Varian Medical Systems, Palo Alto, CA) was implemented. After the accomplishment of the matching procedures, the final correction parameters were automatically applied to the treatment couch and the patient was subsequently treated.

All patients received ADT (LHRH agonist only), which typically started 3–4 months before RT and continued for a total duration of ≥6 months. All patients received ADT concurrent with pelvic RT.

### Follow-up

Acute GU and GI toxicity was evaluated using the Radiation Therapy Oncology Group/European Organization for Research and Treatment of Cancer (RTOG-EORTC) system, extended by additional symptoms [17]. Patients were evaluated weekly during 12 weeks starting from the beginning of irradiation.

Patient-reported QOL outcomes were collected at the baseline and then monthly during acute period of treatment. The EPIC questionnaire with validated 50 items for measurement of prostate cancer specific health related QOL was used [18].

To date only data on acute toxicity and QOL during the acute period of treatment of the first 124 patients are presented in this interim analysis.

### Statistical analysis

Two-sample t tests were used to assess differences between the dosimetric parameters and GU and GI toxicity between the treatment arms. Confirmatory analyses were performed using nonparametric Wilcoxon tests. Stepwise ordinal logistic regression modeling was used to determine independent predictors of GU and GI toxicity. The variables for the acute toxicity were GU and GI grade ≥ 1, ≥ 2 and ≥ 3. Covariates included: rectal volume, bladder volume, rectal and bladder dose/volume cut-points (V40 Gy/V33 Gy, V50 Gy/V42 Gy, V60 Gy/V50 Gy, V70 Gy/V58 Gy, and V76 Gy/V63 Gy) and treatment group (Arm 1 vs. Arm 2).

The baseline EPIC scores (T0) were collected before the start of the treatment. The T1 scores reflects the change in QOL during first 4 weeks of treatment, T2 – during week 5 to 8, T3 – week 9 to 12. The baseline EPIC score was the mean score of all patients from both treatment arms. A simple subtraction of the baseline score (T0) from the subsequent time points scores (T1-3) was calculated for each patient. The average changes from the baseline score in each EPIC score were then compared between treatment arms at each time point using two-sample t-test, with the level of significance of *p* < 0.05. The one-way analysis of variance (ANOVA) for repeated measurements of EPIC mean scores between treatment arms was also performed. A clinically relevant change (CRD) in the quality of life defined as a difference from baseline (T0) to follow-up (T1-3) that exceeded half a standard deviation of the baseline value at month 1, 2 and 3 was compared among the study arms using two-sample t test. The analyses were conducted using StatView (SAS Institute Inc. USA).

## Results

All 124 patients completed the planned radiotherapy (Arm 1–57, Arm 2–67) and were followed for at least for 3 months from the beginning of irradiation (median – 7, range, 3–12 months). There were no protocol violations in terms of inclusion criteria and dose/volume restrictions of treatment plans. Patient compliance was good reaching 93% (53 patients) in Arm 1 and 88% (59 patients) in Arm 2 at 3 months follow-up. The pretreatment characteristics and dosimetric parameters are outlined in Table 1. The recorded characteristics of age, Gleason score, cT stage, and iPSA level appeared to be balanced between both study arms. OAR dosimetric characteristics are V_52.6%_, V_65.8%_, V_78.9%_, V_92%_ and V_100%_ which represents bladder and rectum volumes receiving 40 Gy and 33 Gy, 50 Gy and 42 Gy, 60 and 50 Gy, 70 Gy and 58 Gy, 76 and 63 Gy for Arm 1 and Arm 2, respectively. We found statistically higher volume percentages of the rectum treated to more than both the high (V_100%_, V_78.9%_) and low (V_52.6%_, V_65.8%_) dose cut-points in Arm 2, as compared with Arm 1. A similar pattern was observed for the bladder V_100%_.

**Table 1:** Patients characteristics and dosimetric parameters

	**Arm 1**	**Arm 2**
N	57	67
Age (years)		
Median (range)	64 (51–75)	66 (50–76)
Gleason score		
≤7	49 (86%)	64 (95%)
>7	8 (14%)	3 (5%)
cT-stage		
<T2c	11 (19%)	12 (18%)
≥T2c	46 (81%)	55 (82%)
iPSA		
≤20 ng/ml	40 (70%)	57 (85%)
>20 ng/ml	17 (30%)	10 (15%)
PTV volume		
PTV_P_ ± SE (cc)	116 ± 35	116 ± 31
PTV_SV_ ± SE (cc)	92 ± 25	92 ± 17
PTV_LN_ ± SE (cc)	608 ± 89	613 ± 102
Bladder		
Total volume ± SE (cc)	139 ± 94	125 ± 60
V_52.6%_ ± SE (cc)	101 ± 54	113 ± 51
V_65.8%_ ± SE (cc)	71 ± 35	79 ± 30
V_78.9%_ ± SE (cc)	47 ± 20	51 ± 19
V_92%_ ± SE (cc)	31 ± 12	34 ± 13
V_100%_ ± SE (cc)*	16 ± 8	20 ± 12
Rectum		
Total volume ± SE (cc)	70 ± 16	69 ± 17
V_52.6%_ ± SE (cc)*	43 ± 12	54 ± 15
V_65.8%_ ± SE (cc)*	30 ± 10	37 ± 12
V_78.9%_ ± SE (cc)*	21 ± 7	24 ± 9
V_92%_ ± SE (cc)	14 ± 5	15 ± 6
V_100%_ ± SE (cc)*	4 ± 4	7 ± 5

*Abbreviations: Arm 1* = conventional fractionation; *Arm 2* = hypofractionation.

*p <0.05 between groups, two-sample t test.

### Acute toxicity

The treatment was well tolerated in both arms with 28% and 25% of patients with no GI toxicity and 18% and 12% experiencing no GU toxicity during and after conventional fractionation (Arm 1) and hypofractionation (Arm 2), respectively. There were no Grade 4 acute GU or GI events and only 7% Grade 3 GU toxicities in both Arms 1 and 2. The maximum acute GU and GI toxicity that was observed weekly during the first 3 months from the start of the treatment is displayed in Table 2. The majority of patients in both Arms 1 and 2 experienced Grade 1–2 GU and GI side effects. The difference between study arms was not statistically significant (*p* > 0.05). At the 3 months from the start of radiotherapy, there were 55% and 85% in Arms 1 and 2 experiencing no GU toxicity; the difference was statistically significant (*p* < 0.05).

**Table 2:** Maximum and week 12 acute GU and GI toxicity

**Group**		** Max GU toxicity**					** Max GI toxicity**		
	**Grade**	**0**	**1**	**2**	**3**	**0**	**1**	**2**	**3**
Arm 1		10 (18%)	31 (54%)	12 (21%)	4 (7%)	16 (28%)	18 (32%)	23 (40%)	0 (0%)
Arm 2		8 (12%)	43 (64%)	11 (16%)	5 (7%)	17 (25%)	24 (36%)	26 (39%)	0 (0%)
Fisher’s test *p*		NS	NS	NS	NS	NS	NS	NS	-
		** GU toxicity at week 12**					**GI toxicity at week 12**		
Arm 1		28 (55%)	21 (41%)	2 (4%)	0 (0%)	36 (71%)	8 (16%)	7 (13%)	0 (0%)
Arm 2		46 (85%)	7 (13%)	1 (2%)	0 (0%)	48 (89%)	4 (7%)	2 (4%)	0 (0%)
Fisher’s test *p*		0.001	0.002	NS	-	0.027	NS	NS	-

*Abbreviations: Arm 1* = conventional fractionation; *Arm 2* = hypofractionation; *NS* = difference not significant (*p* < 0.05).

**Figure 1:**
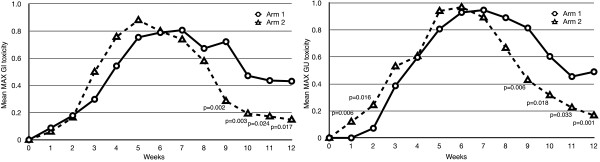
Weekly changes in the mean GU and GI toxicity.

The weekly maximum GU and GI toxicity (mean values) is displayed in Figure 1, demonstrating that there was an increase in the maximum GI toxicity in Arm 2 at weeks 3, 4 and 5 of the treatment, as compared to arm 1. However, those differences between study arms were not significant (*p* > 0.05). The same figure demonstrates a significant decrease in the mean maximum GI toxicity in Arm 2 compared to Arm 1 during week 9, 10, 11 and 12 (*p* < 0.05). In concordance, as compared to Arm 1, there was a significant increase in mean maximum GU toxicity seen in the Arm 2 at week 1 and 2 (*p* = 0.06, *p* = 0.016), but, again, the figure demonstrated a significant decrease of the same toxicity in Arm 2 at week 8, 9, 10, 11 and 12 (*p* < 0.05).

In the multivariate analysis via logistic regression the only covariate shown to be related to increased acute Grade ≥1 GU reactions was the study arm (Arm 1 vs. Arm 2, *p* = 0.049). Neither dosimetric or volume parameters were significant.

### Quality of life

**Figure 2:**
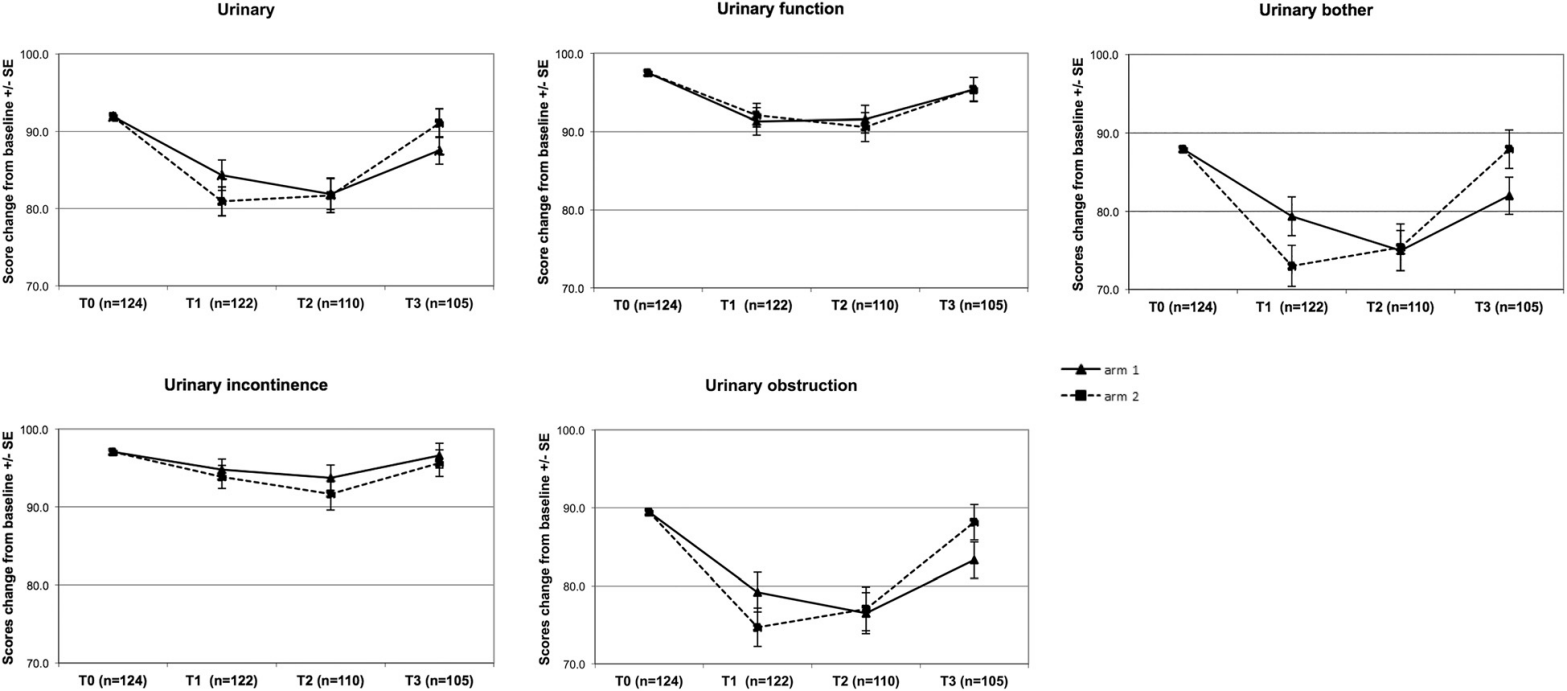
Mean change from baseline ± SE of urinary domain and subscales.

**Figure 3:**
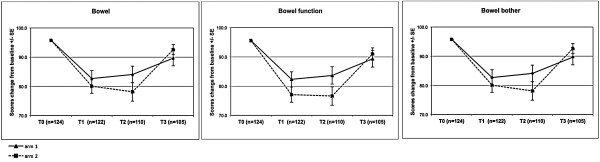
Mean change from baseline ± SE of bowel domain and subscales.

The mean EPIC scores for each domain at baseline and months 1, 2 and 3 were summarized in Table 3. Monthly changes in the mean and SEs for EPIC scores for urinary and bowel domains are displayed in Figure 2 and Figure 3. There are no statistically significant differences of mean EPIC scores in any domain and sub-scales among arms. Overall the worsening of patient-reported urinary and bowel domains is deeper at month 1 and 2 in Arm 2. However, the faster recovery of quality of life parameters at month 3 is observed in the same group of patients.

**Table 3:** EPIC scores (± SE) at each time point

**Time points**	**Baseline**	**1 month**		**2 months**		**3 months**		***p*-value (ANOVA)**
**Arm**	**1/2**	**1**	**2**	**1**	**2**	**1**	**2**	
**N**	**=124**	**=55**	**=67**	**=54**	**=56**	**=51**	**=54**	
Urinary domain	91.9	84.3 (2.0)	81.0 (1.9)	81.9 (2.0)	81.7 (2.2)	87.5 (1.8)	91.1 (1.8)	0.5470
Function	97.5	91.3 (1.7)	92.1 (1.5)	91.6 (18)	90.6 (1.8)	95.3 (1.6)	95.4 (1.5)	0.8058
Bother	87.9	79.4 (2.5)	73.0 (2.6)	75.0 (2.6)	75.4 (3.0)	82.0 (2.4)	87.9 (2.4)	0.4913
Incontinence	97.1	94.8 (1.4)	93.8 (1.5)	93.7 (1.6)	91.7 (2.1)	96.6 (1.6)	95.6 (1.7)	0.8293
Irritative/obstructive	89.5	79.2 (2.6)	74.7 (2.5)	76.5 (2.6)	77.0 (2.8)	83.3 (2.3)	88.1 (2.3)	0.4556
Bowel domain	95.8	82.7 (2.7)	80.1 (2.5)	84.1 (2.8)	78.1 (3.2)	89.7 (2.6)	92.6 (1.7)	0.9208
Function	95.6	82.3 (2.6)	77.1 (2.6)	83.7 (3.0)	76.6 (3.2)	89.3 (2.8)	91.0 (2.1)	0.7569
Bother	95.8	82.7 (2.7)	80.1 (2.5)	84.1 (2.8)	78.1 (3.2)	89.7 (2.6)	92.6 (1.7)	0.9208
**N**	**=121**	**=53**	**=66**	**=53**	**=55**	**=50**	**=53**	
Sexual domain	28.6	22.1 (2.4)	22.0 (2.9)	26.1 (3.0)	26.1 (3.1)	23.2 (3.2)	24.2 (3.4)	0.3919
Function	16.0	8.6 (2.8)	8.1 (3.2)	11.1 (3.2)	11.3 (3.4)	9.5 (3.3)	10.4 (3.9)	0.6457
Bother	56.9	52.4 (5.0)	53.2 (4.9)	59.9 (5.9)	59.4 (6.3)	54.0 (5.7)	55.3 (6.0)	0.3805
Hormonal domain	83.9	83.6 (1.8)	84.7 (1.3)	86.2 (1.8)	83.5 (2.0)	85.1 (2.2)	86.5 (1.9)	0.9304
Function	75.9	76.0 (2.7)	77.7 (2.4)	79.0 (2.6)	75.4 (3.6)	77.3 (2.9)	80.6 (2.6)	0.9012
Bother	90.5	90.0 (1.3)	90.5 (1.1)	92.3 (1.5)	90.2 (1.6)	91.7 (2.0)	91.5 (2.0)	0.7273

*Abbreviations: Arm 1* = conventional fractionation; *Arm 2* = hypofractionation; N = number of patients.

The proportion of patients with CRD of EPIC scores in the urinary and bowel domains are summarized in Table 4. The CRD in urinary domain was significantly higher in Arm 2 at month 1. The proportion of the same group of patients was significantly lower at month 3 from the start of treatment. The proportions of CRD in quality of life related to bowel toxicity were not significant between study arms.

**Table 4:** Clinically relevant decrease (CRD) in EPIC urinary and bowel domains

**Group**	** Urinary domain**			** Bowel domain**		
	**Month**	**Month**	**Month**	**Month**	**Month**	**Month**
	**1**	**2**	**3**	**1**	**2**	**3**
Arm 1	40%	59%	37%	53%	48%	29%
Arm 2	57%	54%	20%	66%	61%	28%
t-test	p = 0.045	NS	p = 0.002	NS	NS	NS

*Abbreviations: Arm 1* = conventional fractionation; *Arm 2* = hypofractionation; *NS* = difference not significant (*p* < 0.05).

## Discussion

The published data for hypofractionation schedules have had a large variability in the treatment regimens, with a range of 5–28 fractions delivered within 3– 5.5 weeks [19,20]. Data on toxicity are controversial and sparse. A few studies have reported the results using high-dose “prostate-only” hypofractionated regimens for the treatment of localized prostate cancer. The acute Grade 2 or greater GI and GU toxicity rates reported in these series ranged from 2% to 35% and 8% to 47%, respectively [3,21].

The acute effects observed for the patients treated herein are in concordance with the studies applying “whole pelvis” IMRT, although there were some differences. We describe about a 40% rate of Grade 2 or higher maximum gastrointestinal reactions in both treatment arms, whereas the rates of other institutional reports are ranging from 0% to 80%, depending on clinical assessment and criteria used [22,23]. The drop in Grade 2 or higher acute GI toxicity to 13% and 4% in our study Arm 1 and Arm 2, respectively, at 3 months from the start of radiotherapy is noteworthy.

In terms of Grade 2 or higher GU reactions, the rates reported from “whole pelvis” IMRT studies are 37% to 60% [22,23]. Therefore, our findings of 28% and 23% in the Arm 1 and Arm 2 respectively are at the lower end. More importantly, by 3 months from the start of radiotherapy, only 1 patient (2%) in Arm 1 and 2 patients (4%) in Arm 2 experienced still Grade 2 GU toxicity.

Compared to Arm 1, GI and GU toxicity both developed significantly earlier but also disappeared earlier in Arm 2. Furthermore, in patients experiencing Grade 2 or greater toxicity, no difference was found in the maximum GI and GU toxicity during 3 months from the start of treatment.

Based on our data, we were not able to define clear risk factors for developing acute GU or GI toxicity after Arm 1 and Arm 2 regimens. In our study the pelvic lymph node irradiation was comparable in both treatment arms (Arm 1 – 23 x 2.0 Gy, Arm 2 – 20 x 2.2 Gy). We believe it plays key role in the development of acute GI toxicity.

It is worth noting that the pre-sacral lymph nodes were generally not irradiated in the majority of “whole pelvis” IMRT series [24]. The inclusion of pre-sacral lymph nodes recently recommended by RTOG guidelines [16] possibly had an impact on acute GI toxicity rate in our study.

We assessed the impact of our Arm 1 and Arm 2 regimens on urinary, rectal, and sexual QOL. Although the results from many prospective studies of hypofractionated RT have been reported, almost all have used only physician-rated toxicity. Hypofractionated RT studies with QOL endpoints are sparse with patients completing EPIC questionnaires after at least 6 months follow-up. The results using this questionnaire during acute period of treatment were reported in two series [25,26]. First prospective study reported QOL outcomes with EPIC questionnaires completed at the baseline and at months 2, 6, 12 and 24. Our Arm 1 regimen seems to be similar to conventional radiation outcomes from this series with an early bowel-related decrease in QOL related to irritation at month 2 [25]. The lack of QOL data at months 1 and 3 makes the comparison with this series difficult. Second phase 1 “prostate-only” dose escalation trial used EPIC questionnaires at the baseline, month 2, 3 and beyond. The mean EPIC scores for bowel and urinary seems to be similar with ours and with the decrease of QOL parameters at month 2 with partial to full recovery at month 3 [26]. Overall we found good correlation between physician-rated GU and GI toxicity and patient-reported QOL outcomes. The deeper worsening of urinary and bowel mean EPIC scores at month 1 and 2 with the faster recovery of those parameters at month 3 is observed in the Arm 2. Those differences were not signifficant. Only the proportion of patients experiencing CRD in urinary domain in Arm 2 was signifficantly higher at month 1 and signifficantly lower at month 3 when comparing with Arm 1.

## Conclusions

In conclusion, there was a small, but significant increase in acute GU reactions at Weeks 1–2 of treatment in the hypofractionation arm. The GI and GU toxicity both developed earlier but recovered faster using hypofractionation. Overall, there was little difference in maximum acute morbidity between the conventional and hypofractionation randomization arms of the IG-IMRT based treatments. Dose–volume criteria were not related to treatment-related increases in acute GI and GU reactions. There was a correlation between acute toxicity and bowel and urinary QOL outcomes. Longer follow-up is needed to determine the significance of these associations with late toxicity.

## Competing interests

The author & co-authors have no conflicts of interest to declare.

## Authors’ contributions

DN, AK created the design of the study, collected the data and wrote the paper. BE, HV performed the statistical analysis, have been involved in drafting the manuscript. RG, MdR, GS, EA, EJ helped to draft the manuscript. KPV participated in the design of the study, have been involved in drafting the manuscript and given final approval of the version to be published. All authors read and approved the final manuscript.
